# Non-Thermal Microbial Inactivation Using Underwater Plasma: Synergistic Effects of Capillary Discharge on *E. coli* and *M. testaceum*

**DOI:** 10.3390/foods14173143

**Published:** 2025-09-08

**Authors:** Eun Jeong Hong, Seungil Park, Seong Bong Kim, Seungmin Ryu

**Affiliations:** Institute of Plasma Technology, Korea Institute of Fusion Energy, 37, Dongjangsan-ro, Gunsan-si, Jeollabuk-do 54004, Republic of Korea; mealie@kfe.re.kr (E.J.H.); spark@kfe.re.kr (S.P.); sbkim@kfe.re.kr (S.B.K.)

**Keywords:** non-thermal, food safety, synergistic effects, underwater plasma, microbial inactivation

## Abstract

This study investigated the synergistic effects of microorganism inactivation using underwater plasma, focusing on applications relevant to food safety. The underwater plasma was generated by capillary electrodes in 10% saltwater circulated at 5 t/h. Two representative bacteria, *Escherichia coli* (Gram-negative) and *Microbacterium testaceum* (Gram-positive), were selected due to their relevance to food and water contamination. Inactivation kinetics were assessed through first-order rate constants (*k*) under direct, indirect, and total (combined) treatments. The rate constant (*k*-value) difference between total and indirect treatment for *E. coli* reached 0.1015 at 5 min of discharge, while *M. testaceum* showed a smaller difference of 0.0083 at 20 min. These results suggest that microorganisms pre-damaged by direct plasma exposure become more susceptible to long-lived reactive species like hydrogen peroxide. The findings indicate that underwater plasma holds significant potential as an effective non-thermal disinfection method for brine solutions, fresh produce, and food-contact surfaces.

## 1. Introduction

With increasing consumer awareness, ensuring microbial safety in the food supply chain has become critical. Microorganisms can contaminate food at various stages—including production, processing, transportation, and storage—posing continuous challenges to food safety and quality [1,2]. Effective disinfection technologies are therefore essential to reduce microbial risks without compromising food integrity.

Traditionally, thermal sterilization techniques have been widely used to address these risks. However, the high temperatures involved can degrade food quality by affecting nutrients, texture, and flavor [3]. Consequently, there is increasing interest in non-thermal processing technologies that can inactivate pathogens while preserving the quality of food [4,5].

Currently, non-thermal sterilization technologies in the food industry comprise physical methods, such as high-voltage pulsed electric fields and ultrasound, as well as chemical methods, including chlorine and ozone. However, pulsed electric fields are limited by their selective action on cell membranes [6], ultrasound exhibits restricted penetration depth [7], chlorine has been associated with the formation of off-flavors and carcinogenic by-products (THM) [8], and ozone is constrained by its short half-life [9]. Plasma differs in that it generates multiple reactive species and physical factors simultaneously, offering broader antimicrobial activity in complex environments such as brine.

In recent years, plasma technology has attracted significant attention for its applications in microbial decontamination [10], food preservation [4,5], and agriculture [11,12]. Unlike conventional treatments, plasma offers both chemical and physical effects in the inactivation of microorganisms without affecting food quality. Research has explored plasma effects on various microorganisms, including bacteria, fungi, viruses, biofilms and bacterial spores. Plasma can be utilized in various environments, including dry gas plasma [13,14], humid gas plasma [15], water surface plasma [16,17], and underwater plasma [18,19].

Underwater plasma treatment has recently gained increasing attention from researchers interested in biological inactivation. This method has proven to be a cost-effective and environmentally friendly technology for inactivating microorganisms in contaminated water. Compared to gas and surface plasma, underwater plasma is generated through direct electrical discharge in the liquid phase, simultaneously producing physical effects such as ultraviolet radiation, strong electric fields, and shock waves, as well as chemical effects including ozone and reactive species, thereby enabling the disinfection of contaminated water [20,21]. It has been shown to effectively inactivate organisms such as *Escherichia coli* and *Bacillus subtilis* without significant altering the pH of the water [22].

While plasma technology has been widely studied for microbial inactivation, few reports have examined the distinct contributions of direct and indirect effects, especially in underwater plasma environments. For instance, Georgescu N. et al. used gas plasma for the inactivation of *S. enterica* and reported that the direct treatment for a relative humidity of 80% at 10 min had similar effects as the indirect treatment at 25 min with humid air [23]. This comparative study of direct and indirect treatments provides a useful reference, but the synergistic mechanisms remain underexplored.

In the production of fermented vegetables such as *kimchi*, a 10% brine solution is typically used during the salting process. However, this brine is often reused, which can lead to the accumulation of microbial contaminants and raise food safety concerns. Since high salt concentrations alone are insufficient to inactivate microorganisms, there is a need for additional sterilization strategies. While plasma treatment has been extensively studied in freshwater or buffer systems, systematic comparisons of its direct, indirect, and total effects in high-salt conditions relevant to food processing have rarely been reported. The synergistic mechanisms of plasma action in brine remain poorly understood, requiring systematic investigation.

This study systematically quantified the synergistic effects of total and indirect underwater plasma treatments in 10% brine, a model condition rarely addressed in previous studies. By clearly distinguishing between direct, indirect, and total treatments in brine, this study provides new insights into their relative contributions and synergistic effects. The findings are expected to contribute to a better understanding of non-thermal disinfection mechanisms in water and support future applications in food hygiene and safety management.

## 2. Materials and Methods

### 2.1. Sample Preparation and Bacterial Culture

A 10% brine solution was used to simulate the salting brine typically applied in *kimchi* preparation, which is often associated with microbial contamination issues. To achieve a salinity of approximately 10%, 10 kg of salt was added to 100 L of tap water (salinity: approximately 10%). *E. coli* and *M. testaceum* were selected as the microorganisms with which to assess the inactivation characteristics. *E. coli* was selected as an indicator organism for Gram-negative bacterium, while *M. testaceum*, was chosen to represent Gram-positive bacteria typically found in salting process.

Both *E. coli* (KCTC 2441) and *M. testaceum* (KCTC 29559) were obtained from the Korean type culture collection (KCTC, Daejeon, Republic of Korea). The *E. coli* colony was inoculated in nutrient broth (Difco, Detroit, MI, USA) and incubated in a shaking incubator at 37 °C and 350 rpm for 24 h. Meanwhile, the *M. testaceum* colony was inoculated in trypticase soy broth (Difco, Detroit, MI, USA) and incubated under the same conditions for 48 h. The initial concentrations of the microorganisms were 7.2 log CFU/mL for *E. coli* and 7.5 log CFU/mL for *M. testaceum*, respectively.

### 2.2. Plasma Reactor Design and Setup

**Figure 1:**
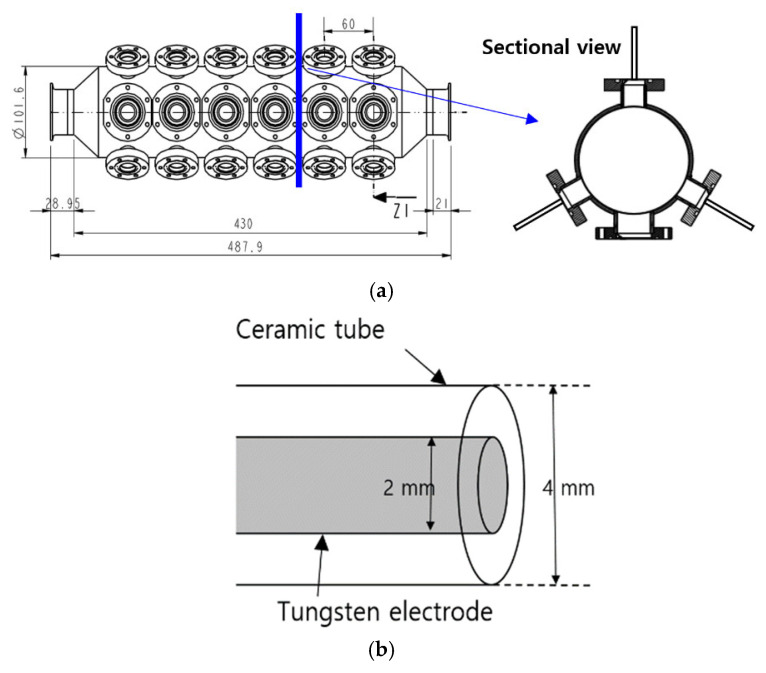

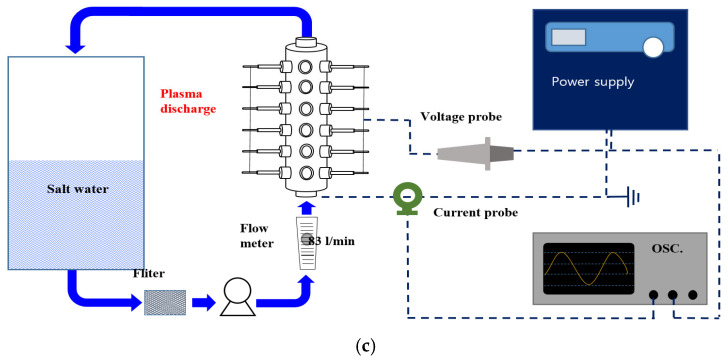
Experimental setup: (**a**) cylindrical type of the plasma reactor, (**b**) capillary electrode, (**c**) underwater plasma system.

Figure 1a shows the cylindrical type of the plasma reactor used in this experiment, which consisted of capillary electrodes, view ports and a body. The body was made of stainless steel with an outer diameter of 101.6 mm, a total length of 487.9 mm, and a thickness of 5 mm. Quartz viewports were installed to observe the optical emission spectroscopy (OES) signals. Eighteen capillary electrodes were inserted into the body. Each electrode was a tungsten rod with a diameter of 2 mm, housed in a ceramic tube with an outer diameter of 4 mm and an inner diameter of 2 mm [24].

The experimental setup included a vessel, a pump, a flow meter and a plasma reactor (Figure 1b). The treatment flow rate of the saltwater was approximately 5 t/h. The plasma was generated by applying AC bi-polar pulsed power (AP150-02-02, EESYS Co., Seongnam, Republic of Korea). Voltage and current signals from the underwater plasma were measured using a high-voltage probe (P6015A, Tektronix, Beaverton, OR, USA) and a current monitor (#110, Pearson Electronics, Palo Alto, CA, USA), respectively. The signals were recorded by a digital oscilloscope (DPO2024, Tektronix).

### 2.3. Plasma Treatment

In general, as plasma discharge times increase, the rate of microorganism inactivation also increases. This effect is considered the direct effect of plasma treatment. A longer contact time between long-lived reactive species produced by underwater plasma and microorganisms enhances the inactivation of microorganisms, representing the indirect effect. Total treatment means a combination of direct and indirect treatments, illustrating a synergistic effect, which can be considered a third state of treatment.

**Figure 2:**
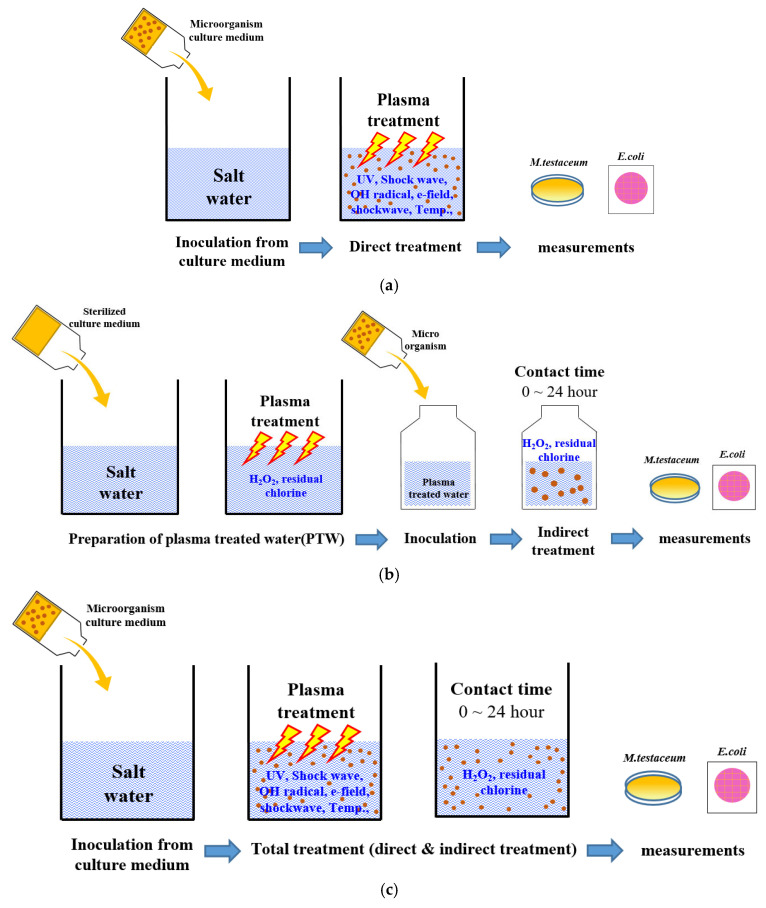
Schematic representation of the plasma treatment: (**a**) direct treatment, (**b**) indirect treatment, (**c**) total treatment.

Figure 2 illustrates the underwater plasma treatment process used in this study. The treatment was divided into direct, indirect and total treatment to analyze the inactivation effects of underwater plasma.

For the direct treatment experiment, microorganisms were inoculated into saltwater at a ratio of 1:200 (microorganism to saltwater) before plasma discharge. The discharge times for *E. coli* were 1, 2, 3, 4, and 5 min, whereas for *M. testaceum,* the times were 5, 10, 15, and 20 min. Samples were taken immediately after the underwater plasma discharge and subjected to microbiological analysis (Figure 2a). For the indirect plasma treatment, a mixture of saltwater and a sterilized culture medium (at a 200:1 ratio) was exposed to underwater plasma discharge. After discharge, the *E. coli* or *M. testaceum* cells were introduced into the plasma-treated water. The samples were subsequently stored at room temperature from 0 to 24 h. Microbiological analysis was immediately performed after the contact time of 0, 1, 3, 6, 12, and 24 h. Total treatment followed the same procedure as the direct treatment but included a contact time of 0 to 24 h after plasma discharge to evaluate the cumulative effects of both direct and indirect interactions. The effectiveness of plasma treatment was evaluated based on the reduction in viable cell counts over time. All experiments were conducted in triplicate, and results are reported as mean ± standard deviation.

### 2.4. Measurement of the Physicochemical Properties

Optical emission spectroscopy (OES) signals were measured to identify reactive species produced during underwater discharge at view port in bottom. The discharge spectrum was measured using a spectrometer (HR4000CG-UV-NIR, Ocean Optics, Inc., Dunedin, FL, USA) with a wide range of 200 to 1100 nm. Due to measurement difficulties, the spectrum was measured at a flow rate of 0 L/min from a single electrode. The temperature was monitored in real-time by a multi-parameter (HI-9829, Hanna Instrument, Seoul, Republic of Korea). The concentration of hydrogen peroxide and free chlorine were measured using titanium sulfate method and DPD method, respectively (HS-H_2_O_2_-L, HS-CL_2_ (Free), HUMAS, Daejeon, Republic of Korea). Color changes were determined using a UV-visible spectrophotometer (HS-3300, HUMAS) according to the manufacturer’s instruction. *H*_2_*O*_2_ only controls were not included. Indirect treatment reflects the role of long-lived ROS, but future studies should include explicit *H*_2_*O*_2_ controls.

### 2.5. Microbiological Measurement

1 mL of the sample solution was diluted in a 10-fold series using 0.9% sterile saline. The diluted *E. coli* solution (1 mL) was then dropped onto 3M Petrifilm coliform count plates (Petrifilm CC, 3M Co., St. Paul, MN, USA). The number of *E. coli* colonies was counted after incubating the plates for one day at 37 °C. For *M. testaceum*, 0.1 mL of the diluted *M. testaceum* solution was dropped onto tryptic soy agar (Difco, Detroit, MI, USA). The number of colonies of the sampled *M. testaceum* was counted after culturing on a solid medium for two days at 37 °C. The number of microorganisms was expressed as colony-forming units (CFU) per mL.

### 2.6. Statistical Analysis

All experiments were performed in triplicate (independent replicates). Microbial inactivation was expressed as *log*_10_(*N_t_*/*N*_0_). Two-way ANOVA (factors: discharge time, contact time, and their interaction) was conducted for each species and treatment (Total, Indirect). Welch’s *t*-test was additionally applied to compare Total vs. Indirect treatments under identical discharge × contact conditions. To control for multiple comparisons, the Benjamini–Hochberg false discovery rate (FDR) was used. Statistical significance was defined at *p* < 0.05. All analyses were performed in Python 3.11 (statsmodels, scipy). For detailed ANOVA and Welch test outputs, see Supplementary Tables S1–S6.

## 3. Results and Discussion: The Process of the Underwater Plasma Treatment

### 3.1. Characteristics of the Underwater Plasma Using a Capillary Electrode

**Figure 3:**
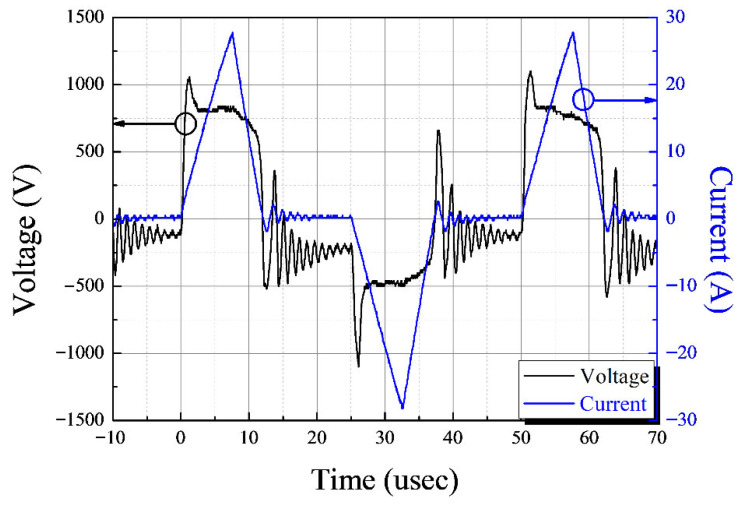
Representative I–V signal during the discharge with 18 capillary electrodes in 10% saltwater.

Figure 3 illustrates the representative voltage and current signal observed during the discharge in 10% saltwater. The peak voltage was approximately 1.1 kV, and the peak current reached about 27 A. The discharge frequency was maintained at 20 kHz, with a pulse width of approximately 10 microseconds. The total power consumption for the underwater treatment of saltwater was calculated to be around 4 kW.

**Figure 4:**
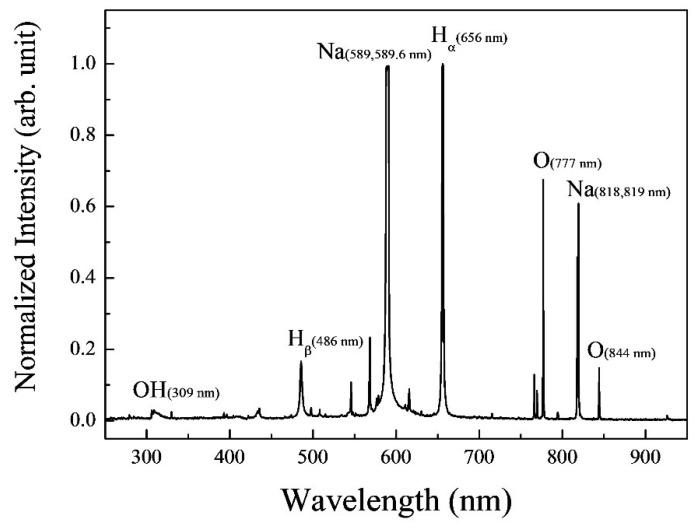
Optical emission spectrum of the underwater plasma in saltwater. Each spectral line corresponds to the chemical species produced from the underwater plasma.

To gather information about the excited species generated by the underwater plasma, optical emission signal (OES) measurements were conducted, as shown in Figure 4. The OES analysis revealed emission signals corresponding to specific reactive species, such as OH radicals (309 nm), hydrogen (Hα: 656 nm, Hβ: 486 nm) and oxygen atom (777 nm and 843 nm) during plasma discharge. Although the OH signal appeared with low intensity due to its short lifetime in aqueous plasma, its presence was confirmed by OES. OH radicals are expected to contribute indirectly to microbial inactivation by forming hydrogen peroxide, which was quantitatively detected as the main long-lived reactive species, such as hydrogen peroxide, which play a key role in microbial inactivation [25,26]. Due to the short lifetime of OH radicals, their quantification was not carried out in this study.

### 3.2. Evaluation of the Physicochemical Properties of the Underwater Plasma

During the plasma discharge in the aqueous phase, several major Reactions (1)–(6) are involved in the production of OH radicals, O radicals and H radicals [25,26].

(1) $$H_{2}O↔OH+H$$

(2) $$2H_{2}O↔H_{2}O^{+}+e_{eq}^{-}+OH\cdot +\;H$$

(3) $$2H_{2}O+e^{-}↔{2H}_{2}O^{*}+e_{eq}^{-}$$

(4) $$H_{2}O^{*}+H_{2}O↔H_{2}O+OH\cdot +\;H$$

(5) $$H_{2}O^{*}+H_{2}O↔H_{2}+O\cdot +\;H_{2}O$$

(6) $$H_{2}O^{*}+H_{2}O↔2H\cdot +\;O\cdot H_{2}O$$

Because these radical species are highly unstable, they react with other molecules in a short time. OH and H radicals could react with each other Equations (7)–(9) to form hydrogen peroxide, water molecules and hydrogen gas [27]. Notably, when underwater plasma is applied to a sodium chloride solution, residual chlorine can be formed through the reaction Equations (10) and (11) [21].

(7) $$OH\cdot +OH\cdot \to H_{2}O_{2}$$

(8) $$OH\cdot +H\cdot \to H_{2}O$$

(9) $$H\cdot +H\cdot \to H_{2}$$

(10) $${Cl}^{-}\to Cl\cdot +e^{-}$$

(11) Cl·+ OH·→HOCl

**Figure 5:**
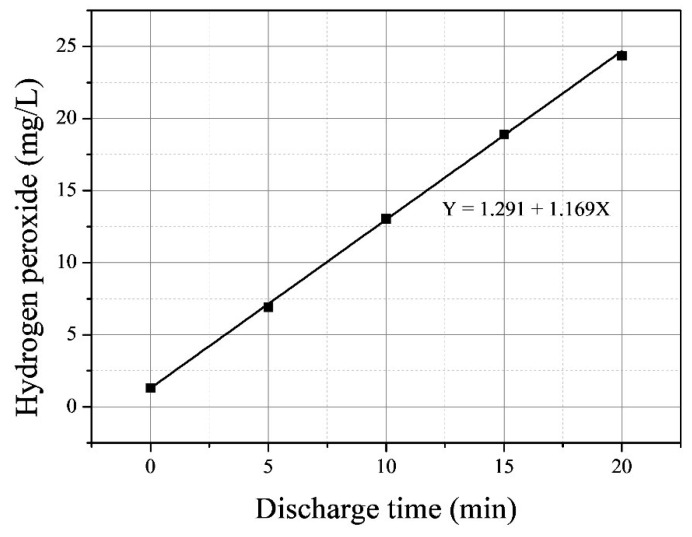
Concentration of long-lived reactive species in relation to discharge time.

During the discharge, we observed the production of *H*_2_*O*_2_ shown in Figure 5. A linear correlation was observed between hydrogen peroxide concentration and discharge time, with a slope of 1.291, indicating a constant rate of production. After 20 min of discharge, hydrogen peroxide concentrations reached 24 ± 0.28 mg/L. Among long-lived ROS, only hydrogen peroxide was detected, while free chlorine was not observed. Other researchers have similarly been unable to measure free chlorine suggesting that active chlorine quickly reacted with hydrogen peroxide [28]. Our findings indicate that hydrogen peroxide is the primary long-term reactive species in plasma-treated water.

During the 20 min plasma discharge, the water temperature increased uniformly from 26 °C to 46 °C. This temperature rise was attributed to joule heating induced by underwater plasma, consistent with the findings by Hong et al. [24]. Table 1 outlines the characteristics of the target water. Temperature is a critical factor affecting the inactivation of microorganisms. According to J. Lee & Kaletunç (2002) and Stringer, George, & Peck (2000), *E. coli* cells are typically inactivated within a temperature range of 50 to 70 °C [29,30]. Therefore, the temperature change in the brine caused by plasma discharge remained below the thermal inactivation threshold for bacteria, indicating that heating was not responsible for microbial reduction.

The pH decreased slightly from 7.1 to 6.8 during the 20 min plasma discharge, but this was insufficient to account for microbial inactivation, which was primarily attributed to reactive species.

### 3.3. Effect of the Underwater Plasma Treatment on the Microorganisms

Table 2 and Table 3 present data on the effects of increasing underwater plasma discharge durations and post-discharge contact times on the population of *E. coli*, assessed through total and indirect treatments, respectively. In Table 2, direct treatment is considered equivalent to a contact time of zero hours. A significant reduction in colony-forming units (CFU) was observed after a plasma discharge of 4 min. This indicates that a specific discharge duration is necessary to achieve effective inactivation, although the required duration varies depending on the type of microorganism. In contrast to direct treatment, total treatment evaluates microorganism inactivation over the entire process, from the time of plasma discharge to the end of the contact period. As such, total treatment requires simultaneous consideration of both discharge time and contact time to fully assess its effectiveness. Complete inactivation of *E. coli* was achieved with a total treatment involving a 3 min plasma discharge and a 12 h contact time, demonstrating that even short discharge durations can guarantee inactivation if sufficient contact time is provided. In contrast, the results for indirect treatment generally exhibited lower inactivation efficacy compared to total treatment (Table 3). After 24 h of contact time, the population of *E. coli* treated with a 3 min plasma discharge showed approximately a 6-log reduction, indicating nearly all microorganisms were inactivated. These results meet the FDA performance standards for microbial safety.

Table 4 and Table 5 show the CFU results demonstrating the effects on *M. testaceum* with increasing underwater plasma discharge times and contact times under total and indirect treatment conditions, respectively. The inactivation effect of direct treatment became evident only after 20 min of plasma exposure. Complete inactivation of *M. testaceum* was achieved with a discharge time of 15 min and a contact time of 24 h under total treatment conditions (Table 4).

Under indirect treatment conditions, complete inactivation of *M. testaceum* was not achieved, even after a discharge time of 20 min and a contact time of 24 h. However, the population was reduced by 2.1 log (Table 5).

While the plasma discharge directly influences microorganism inactivation, the extended contact time with plasma-treated water contributes significantly to the total treatment effect. This effect is particularly pronounced in indirect treatment, where the inactivation process is driven by long-lived reactive species, such as hydrogen peroxide, rather than direct plasma exposure.

The enhanced inactivation observed in total treatment is attributed to the combined effects of initial direct plasma exposure and the subsequent action of long-lived reactive species. During plasma discharge, short-lived reactive species such as OH radicals and atomic oxygen are produced and can rapidly transform into longer-lived species like hydrogen peroxide within the aqueous environment. These species continue to inactivate microorganisms over time, even after the plasma discharge has ceased. This synergy—where direct damage weakens microbial cells and facilitates further inactivation by long-lived species—is the basis of the total treatment effect.

### 3.4. Effect Factors of the Underwater Plasma Inactivation in Saltwater

**Figure 6:**
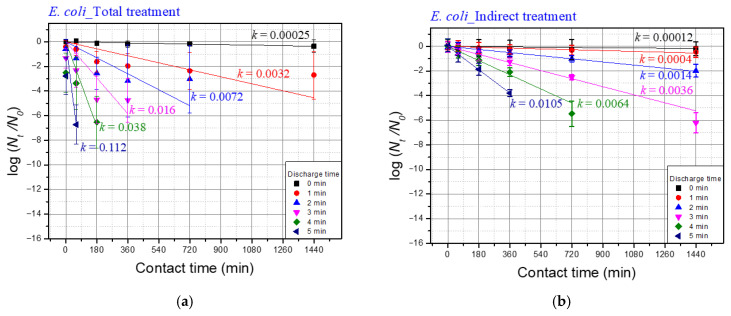
Effects of a plasma treatment on *E. coli* in saltwater (**a**) inactivation effect of the total treatment, and (**b**) inactivation effect of the indirect treatment.

**Figure 7:**
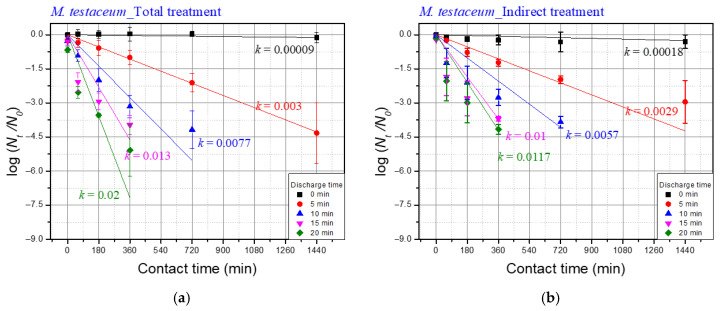
Effects of a plasma treatment on *M. testaceum* in saltwater (**a**) inactivation effect of the total treatment, and (**b**) inactivation effect of the indirect treatment.

Figure 6 and Figure 7 illustrate the normalized inactivation kinetics of microorganisms treated with plasma discharge. The inactivation model used to interpret the results of this study followed first-order reaction kinetics based on Chick’s law, expressed as:

(12) $$\log\left(\frac{N_{t}}{N_{0}}\right)=\;-kt$$

Here, *N*_0_ is the initial number of microorganisms (CFU/mL), *N_t_* is the number of microorganisms at the contact time *t* (h), *k* is the first-order rate constant (min^−1^), and *t* is the contact time (min). In order to isolate the effect of the plasma treatment on the inactivation of the target microorganisms, the values of the reaction constant *k* were analyzed after the raw data was normalized. All experiments were performed in triplicate. For pairwise comparisons between total and indirect treatments at 24 h, Welch’s *t*-tests were conducted on log(*N*_24_/*N*_0_) values (two-sided, α = 0.05).

The *k* value varies depending on multiple factors, including the type of microorganism and the concentration of the disinfectant. Herbert Watson expanded on this concept by modifying *k* to *k′C^n^*, where *C* represents the concentration of the disinfectant. In this study, the concentration of hydrogen peroxide was observed to increase with discharge time, suggesting that the *k* value should also increase proportionally with discharge time. Differences in the *k* values between total treatment and indirect treatment may indicate changes in the state of the microorganisms. This analysis aims to verify the synergistic effects associated with total discharge.

Figure 6a shows the *k* values for *E. coli* under total treatment conditions for discharge times of 1, 2, 3, 4, and 5 min, while Figure 6b presents the *k* values for indirect treatment. Total treatment showed significantly higher *k* values, indicating a faster inactivation rate compared to indirect treatment. This difference is likely due to synergistic effects, which amplify the inactivation process. Synergistic effects were evident in the steeper slope of the inactivation curve for total treatment. This aligns with the findings of Lamei Li et al. [31], who proposed that plasma-treated microorganisms transition through an intermediate state before complete inactivation. The direct damage caused by direct treatment by-products, such as short-lived reactive species, UV, shock wave, weakens the cell structure, making it more susceptible to long-lived reactive species like *H*_2_*O*_2_ during the contact period.

The *k* values for *M. testaceum* under total treatment conditions after 5, 10, 15, and 20 min are depicted in Figure 7a, while Figure 7b shows the effects of indirect treatment. Unlike *E. coli*, the *k* values for *M.testaceum* did not differ significantly between total and indirect treatments. This outcome reflects the structural differences between Gram-negative and Gram-positive bacteria. The thick peptidoglycan layer in *M. testaceum* likely reduces its susceptibility to the synergistic effects observed in *E. coli* [32,33].

Table 6 presents the difference in *k*-values between total treatment and indirect treatment. For *E. coli*, the difference was 0.1015 at a discharge time of 5 min, whereas for *M. testaceum*, the difference was 0.0083 at a discharge time of 20 min. Generally, the difference in *k*-values increased with longer discharge times for both bacterial species, though the trend was more pronounced in *E. coli* than in *M. testaceum*.

This difference in *k*-values quantifies the synergistic effect of underwater plasma treatment. A higher difference indicates that microorganisms exposed to total treatment experienced additional inactivation compared to indirect treatment alone. This supports the hypothesis that direct plasma exposure weakens microbial cells, increasing their susceptibility to long-lived reactive species such as *H*_2_*O*_2_.

Two-way ANOVA on the *log*(*N_t_*/*N*_0_) dataset (Figure 6 and Figure 7) showed that discharge time, contact time, and their interaction were all highly significant for both *E. coli* and *M. testaceum* (*p* < 0.05; see Supplementary Tables S1–S6). Welch’s *t*-tests identified a small number of significant differences between Total and Indirect treatments under matched conditions (*E. coli*: 3/24, *M. testaceum*: 1/25), but none remained significant after FDR correction. Thus, the emphasis is placed on the ANOVA-level effects and overall trends rather than isolated pairwise differences.

**Figure 8:**
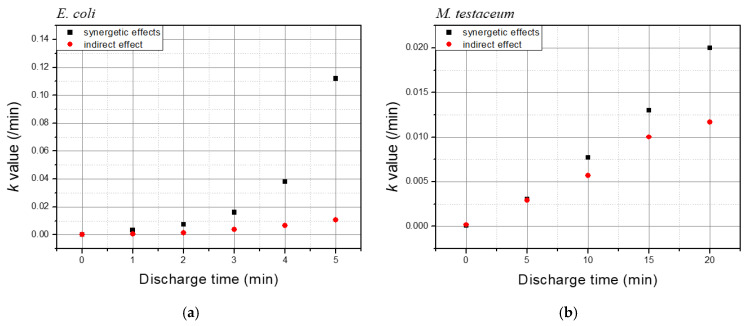
Inactivation rate constant of microorganisms versus the discharge time of the plasma (**a**) *E.coli* (**b**) *M. testaceum*.

In this experiment, the concentration of hydrogen peroxide increased linearly with discharge time. Consequently, if microbial inactivation is primarily driven by ROS concentration, the *k*-value should also exhibit a linear increase with discharge time (Figure 5). As shown in Figure 8, the *k*-values for *E. coli* and *M. testaceum* under indirect treatment generally follow a linear trend. However, the *k*-values of total treatment associated with the synergistic effect in *E. coli* exhibit an exponential increase rather than a linear one. This suggests that beyond a certain threshold, *E. coli* becomes increasingly sensitive to reactive species, accelerating the inactivation rate. In contrast, *M. testaceum* has not yet demonstrated this exponential trend within the 20 min discharge window tested in this study. However, if the discharge time exceeds 20 min, the cells are expected to undergo greater structural damage due to prolonged exposure to reactive species, leading to an increased synergistic effect.

**Figure 9:**
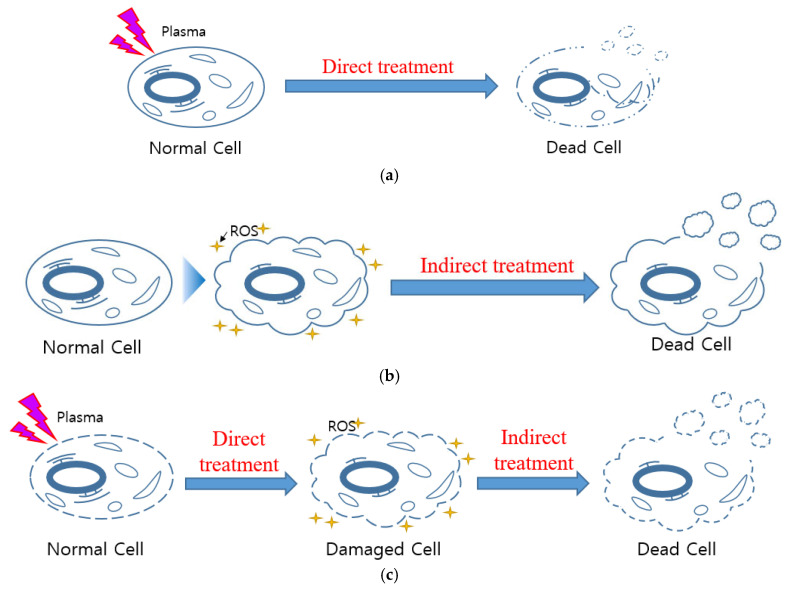
The schematic representation for the inactivation effect of the underwater plasma treatment of microorganisms: (**a**) Direct effect (**b**) Indirect effect (**c**) Synergistic effect.

Figure 9 illustrates the effects of plasma inactivation in saltwater across three treatment groups: direct, indirect, and synergistic effects.

Figure 9a depicts the direct effects of physicochemical factors caused by plasma, including UV radiation, shock waves, electric fields, temperature changes, and short-lived reactive species. These factors directly damage microorganisms, resulting in immediate inactivation. Figure 9b represents the indirect effects of chemical treatment driven by plasma-generated long-lived reactive species, such as *H*_2_*O*_2_. These species persist in the water and continue to inactivate microorganisms over time. The kinetic constant for this effect is influenced by discharge time, reflecting the accumulation of residual active species. Figure 9c highlights the synergistic effect, where microorganisms initially damaged by direct plasma effects are further inactivated by indirect effects. This enhanced inactivation occurs even though the concentration of active species like *H*_2_*O*_2_ being similar in both total and indirect treatments. The key difference lies in the state of microorganisms; those exposed to direct effects are more susceptible to subsequent inactivation by long-lived species. In summary, microorganisms are first damaged by direct effects and then further inactivated by indirect effects. This observation is similar to the results reported by Z.Zhang et al. [34].

## 4. Conclusions

This study demonstrated that underwater plasma generated by capillary discharge electrodes effectively inactivates microorganisms through three distinct mechanisms: direct inactivation via physicochemical effects (such as UV radiation and short-lived reactive species), indirect inactivation mediated by long-lived reactive species (such as hydrogen peroxide), and a synergistic mechanism combining both effects.

Experimental results revealed that *E. coli* and *M. testaceum* respond differently to plasma treatment. *E. coli* exhibited a more pronounced synergistic effect, with a *k*-value difference of 0.1 at a 5 min discharge time, while *M. testaceum* showed a smaller difference of 0.008 at 20 min. These differences reflect microbial structural characteristics, particularly the thick peptidoglycan layer of Gram-positive bacteria, which may reduce susceptibility to plasma effects. These trends were statistically validated using the *log*(*N*/*N*_0_) dataset, as presented in the Results section.

These findings highlight the crucial role of microorganism-specific characteristics in determining the efficacy of plasma-based disinfection. The findings of this study contribute to a clearer understanding of the disinfection mechanisms of underwater plasma and suggest its applicability as a non-thermal alternative to conventional heat-based sterilization methods, particularly for the reuse of brine solutions, washing of fresh produce, and disinfection of food-contact surfaces. Future studies should address scale-up validation, ROS stability, energy efficiency, application to food matrices, and testing against additional pathogens and spores.

## Figures and Tables

**Table 1:** Physicochemical properties of treated water after underwater plasma discharge.

Discharge Time	Temp.	pH	Conductivity (uS/cm)
Control	26.1 ± 2.1	7.2 ± 0.3	145.0 ± 5.4
5 min	30.4 ± 2.2	7.2 ± 0.3	154.2 ± 5.5
10 min	35.7 ± 2.2	7.1 ± 0.2	166.0 ± 5.6
15 min	40.7 ± 2.0	6.8 ± 0.2	176.8 ± 6.7
20 min	45.9 ± 2.0	6.8 ± 0.2	187.3 ± 9.5

Data represent the mean ± s.d. obtained from three independent measurements.

**Table 2:** Populations (log CFU/mL) of microorganisms in saltwater after plasma total treatment of *E. coli*.

Contact Time (h)	Discharge Time (min)					
	0	1	2	3	4	5
0	7.5 ± 0.6	7.1 ± 0.1	6.9 ± 0.2	6.2 ± 0.6	5.0 ± 1.8	4.7 ± 1.6
1	7.5 ± 0.6	6.9 ± 0.2	6.1 ± 0.4	5.2 ± 0.8	4.1 ± 1.5	0.7 ± 1.3
3	7.4 ± 0.6	5.9 ± 0.6	4.9 ± 0.7	2.8 ± 1.3	1.0 ± 1.6	
6	7.3 ± 0.4	5.5 ± 0.4	2.6 ± 2.3	0.8 ± 1.3		
12	7.3 ± 0.5	5.1 ± 0.9	1.3 ± 2.3			
24	7.1 ± 0.1	4.8 ± 1.3	1.4 ± 2.4			

All microbial counts are expressed as log (CFU/mL). Negative values or error ranges extending below zero do not represent negative populations but indicate that the microbial counts are below 1 CFU/mL, i.e., below the detection limit (LOD).

**Table 3:** Populations (log CFU/mL) of microorganisms in saltwater after plasma indirect treatment of *E. coli*.

Contact Time (h)	Discharge Time (min)					
	0	1	2	3	4	5
0	6.9 ± 0.6	7.0 ± 0.2	6.9 ± 0.2	6.9 ± 0.2	7.0 ± 0.2	7.0 ± 0.1
1	6.9 ± 0.3	7.0 ± 0.1	6.8 ± 0.2	6.7 ± 0.2	6.5 ± 0.1	6.2 ± 0.1
3	6.8 ± 0.3	6.8 ± 0.1	6.5 ± 0.3	6.1 ± 0.2	5.8 ± 0.1	5.0 ± 0.2
6	6.7 ± 0.3	6.7 ± 0.2	6.4 ± 0.3	5.7 ± 0.4	4.8 ± 0.2	3.1 ± 0.7
12	6.7 ± 0.4	6.6 ± 0.2	5.9 ± 0.3	4.4 ± 0.4	1.4 ± 1.4	
24	6.7 ± 0.2	6.4 ± 0.2	4.9 ± 0.4	0.7 ± 1.2		

All microbial counts are expressed as log (CFU/mL). Negative values or error ranges extending below zero do not represent negative populations but indicate that the microbial counts are below 1 CFU/mL, i.e., below the detection limit (LOD).

**Table 4:** Populations (log CFU/mL) of microorganisms in saltwater after plasma total treatment of *M. testaceum*.

Contact Time (h)	Discharge Time (min)				
	0	5	10	15	20
0	7.5 ± 0.1	7.3 ± 0.1	7.3 ± 0.1	7.2 ± 0.2	6.9 ± 0.02
1	7.6 ± 0.1	7.2 ± 0.1	6.6 ± 0.1	5.5 ± 0.4	5.0 ± 0.2
3	7.6 ± 0.1	7.0 ± 0.2	5.5 ± 0.4	4.6 ± 0.2	4.0 ± 0.1
6	7.6 ± 0.3	6.5 ± 0.2	4.4 ± 0.4	3.6 ± 0.3	2.5 ± 1.0
12	7.6 ± 0.1	5.4 ± 0.3	3.4 ± 0.7	2.0 ± 0.3	1.8 ± 0.2
24	7.4 ± 0.1	3.2 ± 1.2	2.0 ± 0.9		

All microbial counts are expressed as log (CFU/mL). Negative values or error ranges extending below zero do not represent negative populations but indicate that the microbial counts are below 1 CFU/mL, i.e., below the detection limit (LOD).

**Table 5:** Populations (log CFU/mL) of microorganisms in saltwater after plasma indirect treatment of *M. testaceum*.

Contact Time (h)	Discharge Time (min)				
	0	5	10	15	20
0	7.6 ± 0.1	7.5 ± 0.2	7.5 ± 0.1	7.4 ± 0.1	7.4 ± 0.1
1	7.5 ± 0.03	7.3 ± 0.1	6.4 ± 0.7	5.7 ± 0.8	5.6 ± 0.9
3	7.4 ± 0.02	6.8 ± 0.2	5.5 ± 0.8	4.8 ± 0.7	4.6 ± 0.8
6	7.4 ± 0.1	6.4 ± 0.1	4.8 ± 0.3	3.9 ± 0.02	3.4 ± 0.3
12	7.3 ± 0.3	5.6 ± 0.3	3.8 ± 0.3	3.3 ± 0.5	2.7 ± 0.1
24	7.3 ± 0.3	4.6 ± 1.0	3.3 ± 0.3	2.7 ± 0.2	2.1 ± 0.5

All microbial counts are expressed as log (CFU/mL). Negative values or error ranges extending below zero do not represent negative populations but indicate that the microbial counts are below 1 CFU/mL, i.e., below the detection limit (LOD).

**Table 6:** The differences in the kinetic rate constant between total treatment and indirect treatment.

Discharge Time	*k* Values of *E. coli*		
	Total Treatment (A)	Indirect Treatment (B)	Differences b/w Total and Indirect (A–B)
1	0.0032	0.0004	0.0028
2	0.0072	0.0014	0.0058
3	0.016	0.0036	0.0124
4	0.038	0.0064	0.0316
5	0.112	0.0105	0.1015
**Discharge** **Time**	***k* Values of *M. testaceum***		
	**Total Treatment (A)**	**Indirect Treatment (B)**	**Differences b/w Total and Indirect (A–B)**
5	0.003	0.0029	0.0001
10	0.0077	0.0057	0.002
15	0.013	0.01	0.003
20	0.02	0.0117	0.0083

Total treatment: The *k* values of total treatment over contact time; indirect treatment: The *k* values of indirect treatment over contact time; differences b/w total and indirect (A–B): synergetic effect.

## Data Availability

The original contributions presented in this study are included in the article/Supplementary Material. Further inquiries can be directed to the corresponding author(s).

## Supplementary Materials

The following supporting information can be downloaded at: https://www.mdpi.com/article/10.3390/foods14173143/s1. Supplementary Tables S1–S4 provide detailed two-way ANOVA results on *log*(*N_t_*/*N*_0_) data for each species and treatment. Supplementary Tables S5 and S6 present Welch’s *t*-tests comparing Total vs. Indirect treatments under matched discharge × contact conditions, with raw *p*-values and FDR-adjusted q-values.
