# Supplementary material for: Non-Thermal Microbial Inactivation Using Underwater Plasma: Synergistic Effects of Capillary Discharge on E. coli and M. testaceum
Source: Foods. 2025 Sep 8;14(17):3143. doi: 10.3390/foods14173143 (PMC12428648; doi:10.3390/foods14173143)
Supplement: Supplementary file 1 [file foods-14-03143-s001.zip › foods-3813774-supplementary.pdf]

Supplementary Materials:

Table S1. Two-way ANOVA results of log(N/N0) for *E. coli* (Total).

Index	*Sum-sq*	*df*	*F*	*PR (>F)*
C (discharge)	312.5	5	30.1	4.4E-11
C (contact)	212.8	4	25.6	2.7E-08
C(discharge):C(contact)	100.4	20	2.4	1.2E-02
Residual	99.8	48	-	-

Table S2. Two-way ANOVA results of log(N/N0) for *E. coli* (Indirect).

Index	Sum-sq	df	F	PR (>F)
C (discharge)	37.60	5	33.5	1.8E-14
C (contact)	50.2	4	55.8	4.3E-17
C(discharge):C(contact)	52.2	20	11.6	2.1E-13
Residual	13.0	58	-	-

Table S3. Two-way ANOVA results of log(N/N0) for *M. testaceum* (Total).

Index	Sum-sq	df	F	PR (>F)
C (discharge)	129.0	4	80.6	3.6E-21
C (contact)	94.4	4	59.0	2.5E-18
C(discharge):C(contact)	40.4	16	6.3	2.4E-07
Residual	20.0	50	-	-

Table S4. Two-way ANOVA results of log(N/N0) for *M. testaceum* (Indirect).

Index	Sum-sq	df	F	PR (>F)
C (discharge)	78.1	4	107.7	6.3E-24
C (contact)	78.8	4	108.6	5.2E-24
C(discharge):C(contact)	25.3	16	8.7	1.5E-0.9
Residual	9.1	50	-	-

Table S5. Welch's t-test results (Total vs Indirect) for *E. coli.*

**Discharge (min)**	**Contact (hr)**	**n-total**	**n-indirect**	**mean-total**	**sd-total**	**mean-indirect**	**sd-indirect**	**t**	**p**	**q-fdr**
**0**	0	3	3	0.0	0.0	0.0	0.0			
**0**	1	3	3	0.1	0.1	0.0	0.3	0.3	0.8	0.9
**0**	3	3	3	-0.1	0.1	-0.2	0.7	0.1	0.9	1.0
**0**	6	3	3	-0.2	0.3	-0.2	0.7	0.0	1.0	1.0
**0**	12	3	3	-0.2	0.2	-0.2	0.8	0.2	0.9	1.0
**1**	0	3	3	-0.4	0.5	0.1	0.3	-1.3	0.3	0.3
**1**	1	3	3	-0.6	0.5	0.0	0.4	-1.7	0.2	0.2
**1**	3	3	3	-1.6	0.8	-0.1	0.4	-2.8	0.1	0.2
**1**	6	3	3	-2.0	1.0	-0.2	0.4	-2.8	0.1	0.2
**1**	12	3	3	-2.4	1.5	-0.3	0.4	-2.3	0.1	0.2
**2**	0	3	3	-0.6	0.8	-0.1	0.4	-1.0	0.4	0.5
**2**	1	3	3	-1.3	1.0	-0.1	0.4	-2.0	0.2	0.2
**2**	3	3	3	-2.5	1.3	-0.4	0.3	-2.7	0.1	0.2
**2**	6	3	3	-4.8	2.9	-0.5	0.3	-2.5	0.1	0.2
**2**	12	3	3	-6.2	2.7	-1.0	0.2	-3.3	0.1	0.2
**3**	0	3	3	-1.3	0.8	0.0	0.4	-2.4	0.1	0.2
**3**	1	3	3	-2.3	1.4	-0.3	0.4	-2.4	0.1	0.2
**3**	3	3	3	-4.7	1.9	-0.8	0.4	-3.5	0.1	0.2
**3**	6	3	3	-6.7	1.8	-1.2	0.3	-5.3	0.0	0.2
**4**	0	3	3	-2.5	1.6	0.1	0.5	-2.6	0.1	0.2
**4**	1	3	3	-3.4	2.1	-0.5	0.5	-2.3	0.1	0.2
**4**	3	3	3	-6.5	2.1	-1.1	0.4	-4.3	0.0	0.2
**5**	0	3	3	-2.8	1.5	0.1	0.5	-3.1	0.1	0.2

**5**	1	3	3	-6.7	1.6	-0.7	0.6	-6.2	0.0	0.2

Table S6. Welch's t-test results (Total vs Indirect) for *M. testaceum*.

Discharge (min)	Contact (hr)	n-total	n-indirect	mean-total	sd-total	mean-indirect	sd-indirect	t	p	q-fdr
0	0	3	3	0.0	0.0	0.0	0.0			
0	1	3	3	0.0	0.2	-0.1	0.1	1.1	0.4	0.7
0	3	3	3	0.0	0.2	-0.2	0.1	1.7	0.2	0.7
**0**	6	3	3	0.0	0.3	-0.2	0.2	1.3	0.3	0.7
**0**	12	3	3	0.0	0.1	-0.3	0.4	1.3	0.3	0.7
**5**	0	3	3	-0.2	0.1	-0.1	0.1	-2.0	0.1	0.7
**5**	1	3	3	-0.3	0.2	-0.3	0.0	-0.8	0.5	0.7
**5**	3	3	3	-0.6	0.3	-0.8	0.2	0.9	0.4	0.7
**5**	6	3	3	-1.0	0.3	-1.2	0.2	1.1	0.3	0.7
**5**	12	3	3	-2.1	0.4	-2.0	0.2	-0.6	0.6	0.7
**10**	0	3	3	-0.3	0.2	-0.1	0.0	-1.9	0.2	0.7
**10**	1	3	3	-0.9	0.3	-1.2	0.6	0.8	0.5	0.7
**10**	3	3	3	-2.0	0.5	-2.1	0.7	0.2	0.8	0.8
**10**	6	3	3	-3.2	0.5	-2.8	0.4	-1.1	0.3	0.7
**10**	12	3	3	-4.2	0.8	-3.8	0.3	-0.7	0.6	0.7
**15**	0	3	3	-0.3	0.2	-0.2	0.0	-0.6	0.6	0.7
**15**	1	3	3	-2.1	0.4	-1.9	0.8	-0.4	0.7	0.8
**15**	3	3	3	-2.9	0.4	-2.8	0.8	-0.3	0.8	0.8
**15**	6	3	3	-4.0	0.4	-3.7	0.1	-1.1	0.4	0.7
**15**	12	3	3	-5.6	1.8	-4.3	0.4	-1.2	0.3	0.7
**20**	0	3	3	-0.7	0.1	-0.2	0.0	-6.6	0.0	0.5
**20**	1	3	3	-2.5	0.2	-2.0	0.9	-0.9	0.4	0.7
**20**	3	3	3	-3.5	0.1	-3.0	0.9	-1.1	0.4	0.7
**20**	6	3	3	-5.1	1.2	-4.2	0.2	-1.4	0.3	0.7
**20**	12	3	3	-5.8	1.7	-4.9	0.2	-0.9	0.5	0.7
